# Social relationships as predictors of extended employment beyond the pensionable age: a cohort study

**DOI:** 10.1007/s10433-021-00603-z

**Published:** 2021-01-26

**Authors:** M. Kauppi, K. C. Prakash, M. Virtanen, J. Pentti, V. Aalto, T. Oksanen, M. Kivimäki, J. Vahtera, S. Stenholm

**Affiliations:** 1 Finnish Institute of Occupational Health, Turku and Helsinki, Finland; 2 Department of Public Health, University of Turku and Turku University Hospital, Turku, Finland; 3 Centre for Population Health Research, University of Turku and Turku University Hospital, Turku, Finland; 4 School of Educational Sciences and Psychology, University of Eastern Finland, Joensuu, Finland; 5 Faculty of Medicine, University of Helsinki, Helsinki, Finland; 6 Institute of Public Health and Clinical Nutrition, University of Eastern Finland, Kuopio, Finland; 7 Department of Epidemiology and Public Health, University College London, London, UK

**Keywords:** Marital status, Retirement, Social engagement, Social participation, Working beyond retirement

## Abstract

The aim is to examine whether characteristics of social relationships predict extended employment beyond the pensionable age among Finnish public sector workers. The study population consisted of 4014 participants (83% women, age 62.56 ± 1.21) of the Finnish Retirement and Aging Study followed between 2014 and 2019. Extended employment was defined as the difference between actual retirement date and individual age-related pensionable date and classified into three groups: no extension (retired on pensionable age or extended by < 3 months), short extension (3 months–< 1 year), and long extension (≥ 1 year) beyond the pensionable date. Characteristics of social relationships and engagement were assessed 18 months prior to the pensionable date. Social engagement was classified into consumptive social participation, formal social participation, informal social participation, and other social participation. Data were analyzed using multinomial regression analysis. Of total study participants, 17.8% belonged to short- and 16.5% belonged to long-extension group. Adjusted for age, occupational status, self-rated health and depression, and having a working spouse (OR 2.34, 95% CI 1.39–3.95) were associated with long extension of employment beyond the pensionable age when compared to no extension among men. Likewise, among women, living alone (OR 1.60, 95% CI 1.28–2.00), having a working spouse (1.85, 1.39–2.45), and high consumptive (1.32, 1.07–1.65), high formal (1.47, 1.17–1.85), and other social participation (0.79, 0.63–0.98) were associated with long extension. Having a working spouse, living alone, and high consumptive social participation were associated with short extension. Several characteristics of social relationships, such as having a working spouse, living alone, and high frequency of social engagement, predicted an extension of employment beyond the pensionable age.

**Supplementary Information:**

The online version contains supplementary material available at 10.1007/s10433-021-00603-z.

## Introduction

Aging of the population and reducing number of working age people have resulted an old-age dependency ratio of 30% in the EU, meaning that for every person aged 65 and older, only three persons are of working age (Eurostat 2017). To tackle challenges with public expenditure, health care and social security costs, as well as workforce availability, the political aim in many countries is to encourage people to continue working longer. In Finland, for example, a pension reform was implemented in 2005, with a provision of financial advantage to postponing retirement, among those aged 63–65 years (Virtanen et al. 2014). Another pension reform came into effect in 2017 with a standardized provision of 3-month annual rise on retirement age thereafter (Eläketurvakeskus 2017).

Previous studies have identified a range of organizational and psychosocial factors at work as drivers of extended working life. High socioeconomic status (Polvinen et al. 2016; Virtanen et al. 2014), good working conditions (Virtanen et al. 2014), good self-reported health (Scharn et al. 2017; Virtanen et al. 2014), good functional capacity (Scharn et al. 2017; Stafford et al. 2017), and good physical and mental health (Nilsson 2016; van der Zwaan et al. 2019) were reported as facilitators of working beyond retirement age, whereas stressful work environment has been reported as a barrier of extended working life (Carr et al. 2016; Hintsa et al. 2015; Juvani et al. 2014; Laine et al. 2009; Carlstedt et al. 2018). In addition, several other factors, such as spouse’s working status or opinion toward working (de Wind et al. 2018; Gonzales and Nowell 2017; Scharn et al. 2017; Sewdas et al. 2017; Szinovacz et al. 2001), financial responsibilities and difficulties (de Wind et al. 2016; Sewdas et al. 2017; Szinovacz et al. 2001), as well as care obligations (Jacobs et al. 2014), have been reported to be associated with retirement intentions or early exit from work life. Characteristics of social relationships are likewise important for health and well-being of an individual and may affect one’s work ability and career decisions (André-Petersson et al. 2007; Canivet et al. 2013; Cohen 2004; Rugulies et al. 2006; Shields 2006; Sinokki et al. 2009, 2010; Väänänen et al. 2004).

Previous prospective studies assessing how different characteristics of social relationships in private life predict actual extension of employment beyond the pensionable age have shown that those who are single or unmarried, have a working spouse, and who are active in volunteering are more likely to work after retirement (Carr and Kail 2013; de Wind et al. 2016; Gonzales and Nowell 2017). There might be several mechanisms explaining these associations. For example, it has been shown that individual’s retirement preferences may be affected by the opinions and advice or retirement behavior of people in the social network (Feldman 1994; Vermeer et al. 2019). In particular, the opinions and behavior of the spouse and children but also friends and coworkers are important. Therefore, an individual whose spouse is working is also more likely to continue working him/herself. On the other hand, those who are single or unmarried may prioritize the work over leisure. With respect to the association between volunteering and extended working life, it might be that those engaging in different social activities have more interactions with a wider social network of individuals, which in turn can offer resources, such as information exchange, social capital, and social support needed (Carr and Kail 2013; Gonzales and Nowell 2017). Therefore, they could have other activities to keep them socially active. In addition, it has been shown that engagement in volunteering and other social activities associates with better health and reduced depressive symptoms (Carr and Kail 2013; Gonzales and Nowell 2017).

In addition, even though men have been found to extend their employment more often than women (Virtanen et al. 2014), it is unknown whether social relationships are associated with retirement timing differently for men and women. For example, women adopt informal care responsibilities more often than men and may, thus, be less likely to extend their employment. On the other hand, women have lower average pension accrual than men (Eläketurvakeskus 2018) due to lower average income level and more disrupted work career (Eurostat 2018), which may force them to continue working longer in certain cases. Furthermore, it is not known whether characteristics of social relationships are similarly associated with shorter and longer extension of employment after mandatory retirement date.

Participation in working life is shown to positively associate with intensity of social participation in old age (Bukov et al. 2002). Social engagements and social connectedness could moderate the association of socioeconomic status and other factors with retirement timing (Lancee and Radl 2012; Shiba et al. 2017). Further, social interactions and participation are reported to matter in one’s decision regarding decision to retire or continue working (Vermeer et al. 2014) because those engaging in different social activities could have other non-work related activities that keep them active and happy. Therefore, in this prospective cohort study of aging Finnish public sector employees, we examined whether different characteristics of social relationships and social engagement in private life are associated with extension in employment beyond the pensionable age among men and women. The gender-specific analyses were carried out because men tend to extend their employment more often than women do (Virtanen et al. 2014).

## Methods

### Study population

The data of the present study were from the Finnish Retirement and Aging Study (FIREA), an ongoing follow-up study of aging public sector employees in Finland. The study design has been described in detail elsewhere (Stenholm et al. 2020). The eligible population for the FIREA study included all public sector employees whose individual retirement date was between 2014 and 2019 and who were working in one of the 27 municipalities in Southwest Finland or in 9 other cities or 5 hospital districts around Finland in 2012. The eligible participants were contacted 18 months before their individual pensionable date by mailing them a questionnaire (*n* = 10,629). The follow-up questionnaires were sent annually at least four times and participants have responded on average 3.9 (SD 1.0) times to the surveys. By the end of 2019, 6783 (64% of the eligible sample) cohort members had responded to at least one questionnaire and of them 5831 had responded at least twice. Those participants who had answered to the questionnaire at least once before the pensionable date and had reported their actual retirement date or were working beyond the pensionable age (minimum of 1 year) were included in the present study (*n* = 4014). The FIREA study was conducted in line with the Declaration of Helsinki and was approved by the Ethics Committee of Hospital District of Southwest Finland.

### Measurement of actual retirement date

This study utilized two different measures of retirement. First, individual pensionable date refers to the information on individual pensionable date of each respondents, which was obtained from the institute for public sector pensions in Finland. In Finland, the Public Sector Pensions Act regulates the retirement ages of the public sector employees. From 2005 onwards, public sector employees can retire on a statutory basis after aged 63 years but at the latest before the age of 68 years.

The pension ages in some occupations were below 63 years because those public sector employees have chosen to keep their earlier retirement age based on previous pension act (for example: 58 years for practical nurses and 60 years for primary school teachers). The institute for public sector pensions in Finland has calculated the individual pensionable date for each employee accordingly and working beyond that date will accrue pension income level. Second, the participants reported the actual retirement date in survey questionnaires. We defined retirement event as a transition from work to full-time retirement based on survey responses.

### Extended employment

We calculated the difference between the individual pensionable date and the actual retirement date in days and then classified participants in to three categories as following:

1. Those who did not extend their employment or extended it less than three months beyond the pensionable date (no extension: women, *n* = 2212; men, *n* = 422)
2. Those who extended their employment from three months to less than one year (short extension: women, *n* = 578; men, *n* = 138) and
3. Those who extended their employment at least one year (long extension: women, *n* = 523; men, *n* = 141).

### Assessment of social relationships

Information about the characteristics of social relationships was obtained from the first survey, conducted 18 months prior to the individual pensionable date for most participants. Marital status was categorized into married or cohabiting and not married, divorced or widowed. Spouse’s employment status was assessed by asking whether a spouse/partner was working full-time (yes, no). Number of social network ties was assessed by means of social convoy model (Antonucci 1986), which is based on a set of three concentric circles representing different levels of closeness to the focal persons. In the innermost circle, the respondents were asked to indicate with how many people they felt so close that it was hard to imagine life without them. The middle circle referred to those persons who felt not quite that close but still important and, in the outer circle, the respondents added the number of those persons who were not already mentioned, but who were close and important enough to belong to the individual’s personal network. Total number of network ties were determined by summing up the number of persons in all circles, and categorized into 0–10 and at least 11 persons.

Social engagement type and frequency were assessed by eight social participation items, which were classified into four categories using similar concepts as in the Maastricht social participation profile (Mars et al. 2009). (1) *Consumptive social participation* included activity, which was characterized as benefiting from society (including cultural activities, such as visits to theater, movies, concerts, exhibitions; studying; attending church and other religious activities). (2) *Formal social participation* concerned activity, which was regarded as contributing to society (club and societal activities). (3) *Informal social participation* included meeting relatives, friends or neighbors. (4) *Other social participation* included activity which did not clearly fit into other categories and which was potentially more solitary than activities in other categories. This included handwork and collecting hobbies, playing an instrument, singing, photographing, painting, physical activity, and outdoor activities. The respondents indicated the frequency of each activity on a 5-point Likert-scale with the response options: every day or most days of week, once or twice a week, once or twice a month, once or twice a year, more rarely or never. The mean value of the scale was calculated in each of the four categories, and those belonging to the highest quartile were regarded as to have high social engagement frequency in that category, partly in line with a previous study (Shiba et al. 2017).

Informal caregiving was assessed with a question “Do you provide care to a family member or a relative who is unable to take care of him or herself?” with binary response (yes, no). Financial difficulties were based on a question of having had severe financial problems (within the past 12 month or earlier, based on yes/no response) (Kivimäki et al. 2002).

### Assessment of covariates

Information about sex, date of birth, and occupational status was obtained from the register of the pension insurance institute for the municipal sector in Finland. The occupational titles of the last occupation preceding retirement were coded according to the International Standard Classification of Occupations (ISCO) and categorized into three groups: high (ISCO classes 1–2, e.g., teachers, physicians), intermediate (ISCO classes 3–4, e.g., registered nurses, technicians), and low (ISCO classes 5–9, e.g., cleaners, maintenance workers) (Statistics Finland 2001). Information about self-rated health was drawn from the first survey conducted prior to the pensionable date. *Self-rated health* was assessed by asking participant to rate their overall health status on a 5-point scale (1 = Good, 2 = Rather good, 3 = Average, 4 = Rather poor, 5 = Poor). The dichotomized (good: good and rather good; suboptimal: average, rather poor and poor) variable was used in the analysis. Depression was defined based on the self-reported doctor diagnosed depression and was used as a dichotomized variable (no, yes).

### Statistical analysis

Baseline characteristics of the study population are presented as means and standard deviations (SD) for continuous variables and as numbers and percentages for categorical variables separately for women and men. We tested and found interactions between gender and marital status (*p* < 0.0001), financial difficulties (*p* < 0.0001), spouse’s working status (*p* < 0.05), informal care giving (*p* < 0.05), and consumptive and formal social participation (*p* < 0.001) on extended employment. Therefore, the results were stratified by gender. The gender-stratified baseline characteristics were tested by using the Chi-square test for categorical variables and analysis of variance for continuous variables. Multinomial logistic regression analysis was used to examine associations between characteristics of social relationships and short and long extension of employment, using no extension of employment as a reference group. The models were adjusted for age (Model I), and age, occupational status, self-rated health and depression (Model II). These selected covariates have been shown to be associated with both retirement timing and with social participation characteristics and activities (Cohen 2004; Carlstedt et al. 2018; Gonzales and Nowell 2017; Virtanen et al. 2014). As a sensitivity analyses, we used social relationship characteristics as continuous variables to check the consistency of the results with the dichotomous responses. Results are presented as odds ratios (ORs) with their 95% confidence intervals (CIs). Statistical analyses were performed with SAS® software, version 9.4 (SAS Institute Inc., Cary NC, USA).

## Results

**Fig. 1:**
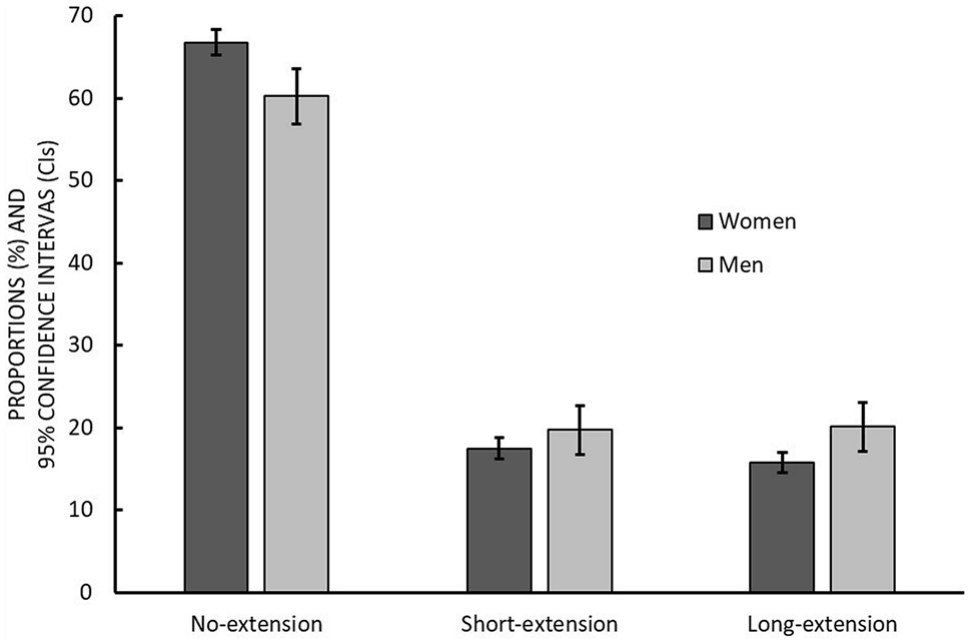
Proportion (with 95% confidence intervals) of no extension (retired on pensionable age or extended by < 3 months), short extension (3 months to < 1 year) and long extension (≥ 1 year) of employment beyond the pensionable age in women (*n* = 3313) and men (*n* = 701)

Average individual pensionable age was 63.8 years among both men and women. Table 1 shows the baseline characteristics for men and women, assessed on average 14 months prior to the individual pensionable date. Men were more likely to be married or cohabiting, to have a working spouse, to be active in consumptive and formal social participation, and to have higher occupational status compared to women. Women, in turn, were more likely to have financial difficulties and larger social networks, to be active in informal and other social participation and to have more often-informal care responsibilities than men. Around one-fourth of women and men had suboptimal self-rated health. More than 80% of the respondents reported they had no depression. Table 2 shows the distribution of covariates among no-extension, short-extension and long-extension groups stratified by gender. Both women and men in high occupational status, with good self-rated health and no depression, were more likely to extend their employment by long term. Figure 1 illustrates distribution of retirement timing for men and women showing that 20% of men and 17% of women extended their working career for a short term, and 20% and 16% for a long term, respectively. The distribution of covariates by social relationship characteristics is presented in Table 1 of ESM.

**Table 1:** Baseline characteristics of the study population by gender

Variables	Total (4014)		Women (*n* = 3313)		Men (*n* = 701)		*p *value^f^
	*n*	(%)	*n*	%	*n*	%	
Age, *Mean (SD)*	62.56 (1.21)		62.55 (1.19)		62.57 (1.32)		0.10
*Occupational status*							< 0.0001
High	1335	33	997	30	338	48	
Medium	1220	31	1083	33	137	20	
Low	1423	36	1202	37	221	32	
*Marital status*							< 0.0001
Married or cohabiting	2774	71	2207	69	567	83	
Not married or cohabiting	1119	29	999	31	120	17	
*Spouse working full-time*^a^							< 0.0001
No	1806	66	1578	72	228	43	
Yes	905	33	599	28	306	57	
*Financial difficulties*							0.004
No	2826	66	2296	72	530	79	
Yes	905	34	872	28	140	21	
*Social relationships*							
Number of members in total social network							0.17
0–10	2657	68	2212	68	445	66	
≥ 11	1248	32	1017	32	231	34	
*Social engagement frequency*							
*Consumptive social participation*^b^							0.24
Low	2497	64	2076	64	421	62	
High	1402	36	1145	36	257	38	
Formal social participation^c^							< 0.0001
Low	2823	73	2396	75	427	63	
High	1034	27	787	25	247	37	
*Informal social participation*^d^							< 0.0001
Low	2545	65	2016	63	529	78	
High	1355	35	1206	37	149	22	
*Other social participation*^e^							< 0.0001
Low	2477	64	1928	60	549	81	
High	1423	36	1294	40	129	19	
*Informal care giving*							0.03
No	3222	82	2639	81	583	85	
Yes	719	18	613	19	106	15	
*Self-rated health*							0.83
Good	2933	74	2428	75	505	74	
Suboptimal	993	25	819	25	174	26	
*Depression*							0.0009
No	3024	85	2476	84	548	89	
Yes	593	15	485	16	68	11	

^a^Those who were not married or cohabiting were excluded

^b^Includes cultural activities, such as visits to theater, movies, concerts, exhibitions; studying; attending church and other religious activities; high = belonging to the highest quartile of the scale

^c^Includes club activity and “non-governmental organization activities”; high = belonging to the highest quartile of the scale

^d^Includes meeting relatives, friends and neighbors¸ high = belonging to the highest quartile of the scale

^e^Includes handwork and collecting hobbies, playing an instrument, singing, photographing, painting, physical activity, outdoor activities; high = belonging to the highest quartile of the scale

^f^Analyses of variance for continuous variables and Chi-square test for categorical variables; SD, standard deviation

**Table 2:** Distribution of covariates among extension groups (no extension: retired on pensionable age or < 3 months after that age, short extension: 3 months to < 1 year and long extension: ≥ 1 year) stratified by gender

Covariates	Total (4014)		Women (*n* = 3313)						*p *value^a^	Men (*n* = 701)						*p *value^a^
			No extension		Short extension		Long extension			No extension		Short extension		Long extension		
	*n*	(%)	*n*	%	*n*	%	*n*	%		*n*	%	*n*	%	*n*	%	
Age (Mean, SD)	62.56 (1.21)		62.58 (1.13)		62.34 (1.26)		62.69 (1.29)		< 0.0001	62.48 (1.37)		62.46 (1.36)		62.95 (1.04)		0.002
*Occupational status*									< 0.0001							< 0.0001
High	1335	33	590	27	203	36	204	39		181	43	62	46	95	67	
Medium	1220	31	718	33	190	33	175	34		89	21	29	21	19	14	
Low	1423	36	888	40	176	31	138	27		149	36	45	33	27	19	
*Self-rated health*									< 0.0001							0.004
Good	2933	74	1548	71	458	81	422	83		303	73	90	67	112	84	
Suboptimal	993	25	624	29	109	19	86	17		108	27	45	33	21	16	
*Depression*									0.94							0.23
No	2993	83	1665	84	425	84	386	83		328	87	113	92	107	91	
Yes	594	17	326	16	81	16	78	17		48	13	10	8	10	9	

^a^Analyses of variance for continuous variables and Chi-square test for categorical variables; SD, standard deviation

The associations between characteristics of social relationships and extended employment beyond the individual pensionable age among *men* are presented in Table 3. Those who had a full-time working spouse were more likely to extend their employment by short (OR 1.71, 95% CI 1.03–2.82) and by long term (OR 2.34, 95% CI 1.39–3.95) when adjusted for age, occupational status, self-rated health and depression (Model II). There was a tendency toward longer extension for those having financial difficulties and high consumptive and formal social participation, but the associations were not statistically significant. Surprisingly, those who had high social participation in other activities, e.g., handwork, playing an instrument, physical activity, or outdoor activities were less likely to extend their employment either by short or long term, but the corresponding estimates were not statistically significant.

**Table 3:** Association between characteristics of social relationships and extended employment (no extension: retired on pensionable age or < 3 months after that age, short extension: 3 months to < 1 year and long extension: ≥ 1 year) beyond the estimated retirement age in men (*n* = 701)

Characteristics of social relationships	No extension (*n* = 422)	Short extension (*n* = 138)				Long extension (*n* = 141)			
		Model I		Model II		Model I		Model II	
	OR (ref.)	OR	95% CI	OR	95% CI	OR	95% CI	OR	95% CI
Married or cohabiting (no vs yes)	1.00	0.93	0.53, 1.61	0.86	0.49, 1.50	0.82	0.46, 1.48	0.89	0.49, 1.62
Spouse working full-time (yes vs no)^a^	1.00	1.67	1.02, 2.74	1.71	1.03, 2.82	2.35	1.41, 3.93	2.34	1.39, 3.95
Financial difficulties (yes vs no)	1.00	1.13	0.69, 1.87	1.18	0.71, 1.98	1.10	0.65, 1.86	1.27	0.73, 2.18
Number of members in total social network (0–10 vs ≥ 11)	1.00	0.89	0.58, 1.37	0.90	0.58, 1.40	1.00	0.64, 1.58	1.09	0.69, 1.73
*Social engagement type and frequency*									
High consumptive social participation^b^ (high vs. low)	1.00	1.01	0.65, 1.58	0.97	0.60, 1.55	1.62	1.05, 2.50	1.28	0.80, 2.03
High formal social participation^c^ (high vs. low)	1.00	1.02	0.66, 1.58	1.07	0.68, 1.68	1.74	1.13, 2.70	1.53	0.98, 2.39
High informal social participation^d^ (high vs. low)	1.00	0.85	0.51, 1.41	0.85	0.51, 1.44	0.96	0.57, 1.62	0.93	0.55, 1.58
High other social participation^e^ (high vs. low)	1.00	0.78	0.45, 1.36	0.78	0.45, 1.38	0.87	0.50, 1.50	0.76	0.44, 1.33
Informal care giving (no vs yes)	1.00	0.82	0.47, 1.46	0.78	0.44, 1.39	0.75	0.41, 1.35	0.77	0.42, 1.41

OR, odds ratio; CI, confidence interval; Model I: Age adjusted; Model II: Adjusted for age, occupational classes, self-rated health and depression

^a^Those who were not married or cohabiting were excluded

^b^Includes cultural activities, such as visits to theater, movies, concerts, exhibitions; studying; attending church and other religious activities; high = belonging to the highest quartile of the scale

^c^Includes club activity and “non-governmental organization activities”; high = belonging to the highest quartile of the scale

^d^Includes meeting relatives, friends and neighbors¸ high = belonging to the highest quartile of the scale

^e^Includes handwork and collecting hobbies, playing an instrument, singing, photographing, painting, physical activity, outdoor activities; high = belonging to the highest quartile of the scale

The association between of social relationship characteristics and extended employment beyond the individual pensionable age among *women* is presented in Table 4. Being unmarried or single (OR 1.60, 95% CI 1.28–2.00), having a spouse working full time (OR 1.85, 95% CI 1.39–2.45), and having financial difficulties (OR 1.90, 95% CI 1.51–2.38) were associated with a long extension of employment beyond the pensionable age, when compared to the no-extension group in model adjusted for age, occupational status, self-rated health and depression (Model II). Likewise, being involved in high consumptive (OR 1.32, 95% CI 1.07–1.65) and high formal social participation (OR 1.47, 95% CI 1.17–1.85) predicted long extension of employment beyond the pensionable age. In addition, those who were unmarried or single (OR 1.28, 95% CI 1.02–1.59), those who had financial difficulties (OR 1.35, 95% CI 1.07–1.70), and those who were involved in high consumptive social participation (OR 1.24, 95% CI 1.00–1.53) were more likely to extend their employment beyond the pensionable age by short term. By contrast, those women who had high social participation in other activities, e.g., handwork, playing an instrument, physical activity, or outdoor activities, were less likely to extend their employment for a long term (OR 0.79, 95% CI 0.63–0.98). As a sensitivity analyses, we examined the social relationship characteristics as continuous variables to examine whether this has an influence on the findings. The results are shown in “Table 2 of ESM,” and the findings were in line with the findings using the social relationship characteristics as dichotomous variables.

**Table 4:** Association between characteristics of social relationships and extended employment (no extension: retired on pensionable age or < 3 months after that age, short extension: 3 months to < 1 year and long extension: ≥ 1 year) beyond the estimated retirement age in women (*n* = 3313)

Characteristics of social relationships	No extension (*n* = 2212)	Short extension (*n* = 578)				Long extension (*n* = 523)			
		Model I		Model II		Model I		Model II	
	OR (ref.)	OR	95% CI	OR	95% CI	OR	95% CI	OR	95% CI
Married or cohabiting (no vs yes)	1.00	1.21	0.97, 1.50	1.28	1.02, 1.59	1.48	1.19, 1.84	1.60	1.28, 2.00
Spouse working full-time (yes vs no)^a^	1.00	1.26	0.96, 1.66	1.18	0.89, 1.56	2.00	1.52, 2.65	1.85	1.39, 2.45
Financial difficulties (yes vs no)	1.00	1.27	1.01, 1.59	1.35	1.07, 1.70	1.78	1.43, 2.22	1.90	1.51, 2.38
Number of members in total social network (0–10 vs ≥ 11)	1.00	0.99	0.79, 1.21	1.04	0.84, 1.30	0.90	0.72, 1.12	0.97	0.77, 1.21
*Social engagement type and frequency*									
High consumptive social participation^b^ (high vs. low)	1.00	1.39	1.13, 1.70	1.24	1.00, 1.53	1.55	1.26, 1.91	1.32	1.07, 1.65
High formal social participation^c^ (high vs. low)	1.00	1.23	0.97, 1.54	1.16	0.92, 1.46	1.59	1.27, 1.99	1.47	1.17, 1.85
High informal social participation^d^ (high vs. low)	1.00	0.92	0.75, 1.13	0.94	0.76, 1.16	0.96	0.77, 1.19	0.99	0.79, 1.23
High other social participation^e^ (high vs. low)	1.00	1.01	0.82, 1.24	1.01	0.82, 1.24	0.78	0.63, 0.97	0.79	0.63, 0.98
Informal care giving (no vs yes)	1.00	0.82	0.64, 1.04	0.83	0.65, 1.07	1.00	0.76, 1.31	1.04	0.79, 1.37

OR, odds ratio; CI, confidence interval; Model I: Age adjusted; Model II: Adjusted for age, occupational classes, self-rated health and depression

^a^Those who were not married or cohabiting were excluded

^b^Includes cultural activities, such as visits to theater, movies, concerts, exhibitions; studying; attending church and other religious activities; high = belonging to the highest quartile of the scale

^c^Includes club activity and “non-governmental organization activities”; high = belonging to the highest quartile of the scale

^d^Includes meeting relatives, friends and neighbors¸ high = belonging to the highest quartile of the scale

^e^Includes handwork and collecting hobbies, playing an instrument, singing, photographing, painting, physical activity, outdoor activities; high = belonging to the highest quartile of the scale

## Discussion

In this prospective study of Finnish public sector employees, several characteristics of social relationships in private life, such as marital status, high social participation, as well as financial difficulties predicted short and long extension of employment beyond the pensionable age. In men and women, having a working spouse was associated with long-term extension. In addition, women who were active in consumptive social participation (e.g., attending cultural and religious activities and studying) or in formal social participation (e.g., being members of club and societal activities) were more likely to extend their employment for at least one year. Interestingly, those women who actively participated in handwork and collecting hobbies, playing an instrument, singing, photographing, painting, physical activity, or outdoor activities, were less likely to extend their employment.

In agreement with previous studies (Gonzales and Nowell 2017; Scharn et al. 2017; Virtanen et al. 2014), we observed that men were more likely than women to extend their employment. It may be that women more often prioritize private-life activities, for example taking care of grandchildren and parents, meeting friends, and sharing time with a spouse when deciding about retirement timing (Szinovacz et al. 2001). Our findings that being alone (Gonzales and Nowell 2017; Virtanen et al. 2014) or having a working spouse was associated with extension of employment accords with previous studies (Gonzales and Nowell 2017; Scharn et al. 2017), and may indicate the principle of ‘linked lives’ (Morrow-Howell et al. 2014). According to that principle, individuals’ lives are bound to the lives of others, and for example, transition to retirement may largely be shaped by close social relationships.

To assess social engagement type and frequency, we used similar concept as in the Maastricht social participation profile with different categories: consumptive social participation, formal social participation, informal social participation, and other social participation, reflecting different types of activities (Mars et al. 2009). To our knowledge, there are no previous longitudinal studies assessing specifically these social activity characteristics with regard to extended employment. We found that women who were active in consumptive social participation (e.g., cultural and religious activities and studying) or in formal social participation (e.g., club and societal activities) were more likely to extend their employment for at least one year beyond the pensionable age. Supporting these results, previous studies have reported an association between volunteering and working after retirement (Carr and Kail 2013; de Wind et al. 2016; Gonzales and Nowell 2017). This suggests that those who are active in leisure time are inclined to actively participate in work life, and vice versa (Carr and Kail 2013; Morrow-Howell et al. 2014). Engaging in different social activities foster also interactions with a wider social network of individuals in heterogeneous social groups, which in turn can offer resources (such as information exchange, social capital, social support) needed to remain active and hold to one’s work (Carr and Kail 2013; Gonzales and Nowell 2017). In addition, engagement in volunteering and other social activities associate with better health and reduced depressive symptoms (Carr and Kail 2013; Gonzales and Nowell 2017), and those, in turn, who have better health are more likely to have more role involvements both in volunteering or other social activities and in work life (de Wind et al. 2016; Morrow-Howell et al. 2014). In our analyses, however, significant association between engagement in social activities and extension of employment remained after adjusting for self-rated health suggesting independent effect of those activities. However, high frequency of social engagement in activities such as handwork and collecting hobbies, playing an instrument, singing, photographing, painting, physical activity, and outdoor activities (listed as other social participation in our study) were associated with a lowered likelihood of long extension of employment among women in our study. There is a lack of previous studies assessing specifically the association of these activities with extension of employment beyond the pensionable age. The study by Tuisku et al. (2016) showed that cultural leisure activities were associated with beneficial recovery experiences and work engagement among hospital employees. These features could be expected to associate with higher likelihood to extend employment. Although we do not have a conclusive explanation to our finding, the difference could be attributed to the type of study. In the aforementioned study, they checked the cross-sectional association compared to the longitudinal predictions used in our study.

In addition to social relationships, we and other studies have observed financial difficulties to predict extension of employment in women (de Wind et al. 2016; Sewdas et al. 2017; Szinovacz et al. 2001). Since women have in general lower average pension accrual than men (Eläketurvakeskus 2018), they may need to continue working for economic reasons more often than men do. However, not all studies have supported the evidence that financial resources are associated with employment after retirement (Dingemans et al. 2016). The reason could be that some people might continue working due to financial difficulties and some other could continue on the same job due to other motivational aspects. On the other hand, it has been suggested that higher income is associated with extended employment (Scharn et al. 2017). We were unable to provide information on those whose financial situations were very good because we did not have information related to income of the participants in our study. Therefore, it is possible that the perception of one’s financial situation is conceptually different from actual value of income, and thus, the results differ (de Wind et al. 2016).

We did not find significant association between care giving and extended employment either in men or in women although previous studies have found that providing high intensity of informal care is associated with higher likelihood of being retired or out of the work force (Bolin et al. 2008; Jacobs et al. 2014). On the other hand, it has been suggested that probability of being employed depends on the type of care provided. Care provided within the household (co-residential care) has been shown to significantly reduce the employment probability, while extra-residential care has not been shown to have an impact on the labor market participation (Heitmueller 2007). In our study, the number of participants who reported to provide informal care giving was small, which may at least partly explain the observed nonsignificant associations. For the same reason, it was not possible to assess whether the association would have been different according to different intensities or type of provided care. On the other hand, providing informal care may be associated with higher likelihood to continue working especially when expenses of care arrangements require that. Moreover, working may provide a coping strategy for dealing with the stress of being a caregiver (Burr et al. 2005).

The use of repetitive yearly measurements among a large representative sample of an occupationally established cohort and date-based information about individual pensionable date and timing of the actual retirement are the strengths of this study. We covered different characteristics of social relationships assessed when still in employment. All participants were still in employment, when first contacted. This ‘healthy worker effect’ implies that those with major health problems, an unlikely population for a lengthy extension of employment, had retired earlier, and were not in our study cohort. Thus, the participants in the groups of no extension, short extension, and long extension were relatively homogeneous suggesting that health related selection bias is not a major concern in our study.

The study has also some limitations, which warrant discussion. First, the lack of information about earlier mid-life experiences, e.g., educational investments, job changes, late transitions into parenthood, or late divorces, which have been shown to associate with intentions to retire later (Damman et al. 2011), is the salient limitation of this study. However, we did have information about occupational and marital status, which reflect some of these earlier experiences. Likewise, we did not take into account the factors associated with early health-based retirement because our sample includes those who continued until old-age pension and retired on their individual pensionable age or worked beyond that. In addition, although our findings may be generalizable to employees in Finland and other Nordic welfare countries, they may not be generalizable to countries with different pension systems. Moreover, there were relatively few men compared to women, due to which not all the associations among men were found statistically significant, despite tending a direction toward an association. This type of gender distribution (83% women in our study) is typical in Finnish public sector occupations (Statistics Finland 2016).

In conclusion, social relationships in private life could predict the choice to extend employment beyond the pensionable age. While having a working spouse was a contributing factor for both sexes, several other factors, such as living alone and high social engagement frequency, were additionally related to extended employment among women. The examination of potential mechanisms through which different characteristics of social relationships contribute to extended employment is warranted for future studies.

## Supplementary Information

Below is the link to the electronic supplementary material.

Supplementary file 1 (PDF 549 KB)

## Data Availability

Not applicable.
