# Supplementary material for: Social relationships as predictors of extended employment beyond the pensionable age: a cohort study
Source: Eur J Ageing. 2021 Jan 26;18(4):491–501. doi: 10.1007/s10433-021-00603-z (PMC8563924; doi:10.1007/s10433-021-00603-z)
Supplement: Supplementary file 1 — (PDF 549 KB) [file 10433_2021_603_MOESM1_ESM.pdf]

Social relationships as predictors of extended employment beyond the pensionable age: a cohort study

Kauppi M*, PhD[1], Prakash KC*, PhD[2,3], Virtanen M, PhD[4], Pentti J, BSc[2,3,5], Aalto V, MSc[1], Oksanen T, MD, PhD[1,6], Kivimäki M, PhD[1,5,7], Vahtera J, MD, PhD[2,3], Stenholm S, PhD[2,3]

Supplement Tables

eTable 1: Distribution of covariates by social relationship characteristics

Covariates	Total (4,014)		Consumptive social participation		Formal social participation		Informal social participation		Other social participation		Social networks		Informal care giving	
			Low% (n=2,497)	High% (n=1,402)	Low% (n=2,823)	High% (n=1,034)	Low% (n=2,545)	High% (n=1,355)	Low% (n=2,477)	High% (n=1,423)	0-10% (n=2,657)	≥11% (n=1,248)	No% (n=3,222)	Yes% (n=719)
	n	%												
Age (Mean, SD)	62.56(1.2)		62.51(1.2)	62.61(1.2)**	62.52(1.2)	62.60(1.2)*	62.56(1.2)	62.52(1.2) ‡	62.52(1.2)	62.59(1.1) ‡	62.55(1.2)	62.52(1.2) ‡	62.59(1.2)	62.42(1.3)***
Occupational-status														
High	1,335	33	25	48***	30	44***	37	28	35	31**	31	40***	33	35‡
Medium	1,220	31	32	29	31	29	29	32	31	30	31	29	31	31
Low	1,423	36	43	24	39	27	34	40	34	39	38	31	36	34
Self-rated health														
Good	2,933	74	73	78***	74	78*	73	77*	72	79***	73	78**	75	76‡
Suboptimal	993	25	27	22	26	22	27	23	28	21	27	22	25	24
Depression														
No	2,993	83	83	84‡	83	84‡	82	84‡	82	85**	82	86**	84	81‡
Yes	594	17	17	16	17	16	18	16	18	15	18	14	16	19

[a]Analyses of variance for continuous variables and Chi-square test for categorical variables; SD, Standard Deviation; ****p*-value<0.0001; ***p*-value<0.01; **p*-value<0.05; ‡*p*-value>0.05

eTable2: Association between characteristics of social relationships (continuous) and extended employment (no extension: retired on pensionable age or <3 months after that age, short-extension: 3 months to <1 year and long-extension: ≥ 1 year) beyond the estimated retirement age in **men** (n=701) and **women** (n=3313)

**Characteristics of social relationships among men**	**No-extension** (n=422)	**Short-extension** (n=138)		**Long-extension** (n=141)	
	OR (ref.)	OR[e]	95% CI	OR[e]	95% CI
Social engagement type and frequency					
Consumptive social participation [a]	1.00	0.89	0.58, 1.37	1.19	0.80, 1.77
Formal social participation [b]	1.00	1.09	0.91, 1.29	1.20	1.01, 1.44
Informal social participation [c]	1.00	1.01	0.79, 1.37	0.82	0.60, 1.13
Other social participation [d]	1.00	1.12	0.94, 1.34	1.02	0.85, 1.23
**Characteristics of social relationships among women**	**No-extension** (n=2212)	**Short-extension** (n=578)		**Long-extension** (n=523)	
	OR (ref.)	OR[e]	95% CI	OR[e]	95% CI
Social engagement type and frequency					
Consumptive social participation [a]	1.00	1.24	1.03, 1.48	1.30	1.08, 1.57
Formal social participation [b]	1.00	1.08	0.98, 1.18	1.19	1.09, 1.31
Informal social participation [c]	1.00	0.89	0.77, 1.03	0.91	0.78, 1.06
Other social participation [d]	1.00	0.99	0.92, 1.08	0.89	0.83, 0.97

[a] includes cultural activities, such as visits to theatre, movies, concerts, exhibitions; studying; attending church and other religious activities

[b] includes club activity and "non-governmental organization activities"

[c] includes meeting relatives, friends and neighbors

[d] includes handwork and collecting hobbies, playing an instrument, singing, photographing, painting, physical activity, outdoor activities

Note: OR, odds ratio; CI, confidence interval

[e]Model Adjusted for age, occupational classes, self-rated health and depression
